# Impact of a syndrome-based stewardship intervention on antipseudomonal beta-lactam use, antimicrobial resistance, *C. difficile* rates, and cost in a safety-net community hospital

**DOI:** 10.1017/ash.2024.28

**Published:** 2024-03-13

**Authors:** Alfredo J. Mena Lora, Rodrigo Burgos, Aaron E Hunt, Yifan Wang, Dylan Huber, Lawrence Sanchez, Mirza Ali, Candice Krill, Eden Takhsh, Susan C. Bleasdale

**Affiliations:** 1 Division of Infectious Diseases, Department of Medicine, University of Illinois at Chicago, Chicago, IL, USA; 2 Saint Anthony Hospital, Chicago, IL, USA

## Abstract

Broad-spectrum antimicrobials are commonly used without indication and contribute to antimicrobial resistance (AMR). We implemented a syndrome-based stewardship intervention in a community hospital that targeted common infectious syndromes and antipseudomonal beta-lactam (APBL) use. Our intervention successfully reduced AMR, *C. difficile* rates, use of APBLs, and cost.

## Introduction

Antimicrobial resistance (AMR) causes over 2 million infections each year in the United States.^1^ Infections with AMR organisms are associated with higher hospitalization rates, length of stay, cost, and mortality.^1^ Improving antimicrobial use (AU) is imperative to curb the development and spread of AMR.^1^ Antimicrobials are commonly overprescribed in hospitals and often given without indications.^2^ Antimicrobial selection is commonly guideline discordant and antipseudomonal beta-lactams (APBLs) frequently used.^3^ Antipseudomonal beta-lactams promote emergence of AMR even with brief exposures.^4,5^ Despite widespread use, empiric selection of APBLs is recommended in limited scenarios. Thus, APBLs can be an important target for antimicrobial stewardship programs (ASPs).

Though accreditation requirements have led to an increase in ASP nationwide, interventions may vary by facility type and capabilities.^6^ Larger hospitals are more likely to have robust ASP, while small hospitals (<200 beds) face implementation challenges such as staffing and lack of infectious diseases (ID) trained pharmacists.^7^ Antimicrobial stewardship program has primarily been studied in academic settings, yet small hospitals and critical access hospitals represent a larger share of hospitals in the United States. Over 70% of hospitals have less than 200 beds, and 10% have less than 25 beds.^8^ Thus, finding effective ASP strategies in these settings is of significance. Proposed changes to The Joint Commission (TJC) standards recommend at least two evidence-based guidelines to improve antibiotic use for the most common indications. We seek to describe the impact of syndrome-based ASP on APBL use in an urban safety-net community hospital.

## Methods

### Study design and setting

We conducted a single-center quasi-experimental study at a 151-bed hospital. Our facility is a safety-net community hospital in the west side of Chicago that provides medical, surgical, pediatric, and obstetrics–gynecology services. Our preintervention period was from October 1, 2016, to September 30, 2017, and our intervention period was from October 1, 2017, to September 30, 2018. Our facility has one ID physician, one lead ASP pharmacist, and four full-time pharmacists. All pharmacists are without ID postgraduate training.

### Intervention

A comprehensive ASP syndrome-based prospective audit and feedback (PAF) targeting APBLs was established. Infectious syndromes addressed by our intervention included skin and soft tissue infections (SSTIs), urinary tract infections (UTIs), community-acquired pneumonia (CAP), pneumonia with AMR risk factors (P-AMR), and complicated intra-abdominal infections (cIAIs). Empiric recommendations were based on IDSA guidelines and adapted to local antibiograms. Guidelines were disseminated via intranet and electronic medical record order sets were created (Supplement A, B (online)). Syndrome-based stewardship training for pharmacists consisted of a modified pharmacist-centered treatment guideline with additional components focusing on definitions of infectious syndromes, severity stratification, empiric drug selection, duration, and de-escalation goals (Supplement C (online)). Education was provided to pharmacists via video modules, paper, pocket guides, and in person. Prospective audit and feedback was implemented on October 1, 2017. Stewardship tasks were divided between shifts.^9^ The midnight shift reviews an automated antimicrobial report and performs chart audits. An accessible online PAF tool is used for documentation. During the day shift, the lead ASP pharmacist reviews the online tool and collaborates with the ID physician for prescriber feedback and just-in-time teaching. Feedback is provided by the pharmacist via phone. Antimicrobial stewardship program required approximately 5 physician hours and 28 pharmacist hours per week.

### Measures

The primary outcome measure was AU, APBL, and non-APBL use. Secondary outcomes included APBL susceptibility for *Pseudomonas aeruginosa* (PSAR), *C. difficile* infection (CDI) rates per 10,000 days, trends in AU by unit, parenteral to oral conversion, and total antimicrobial expenditures. Antimicrobial use was measured as days of therapy per 1,000 patient days (DOT/1000) and was compared before and after implementation.

## Results

**Figure 1.**
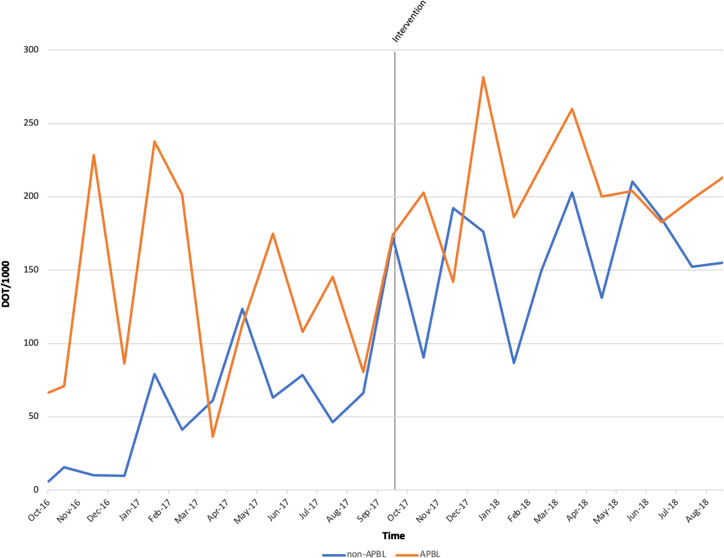
DOT/1000 of APBL and non-APBL in ICU settings by month before and our intervention.

**Figure 2.**
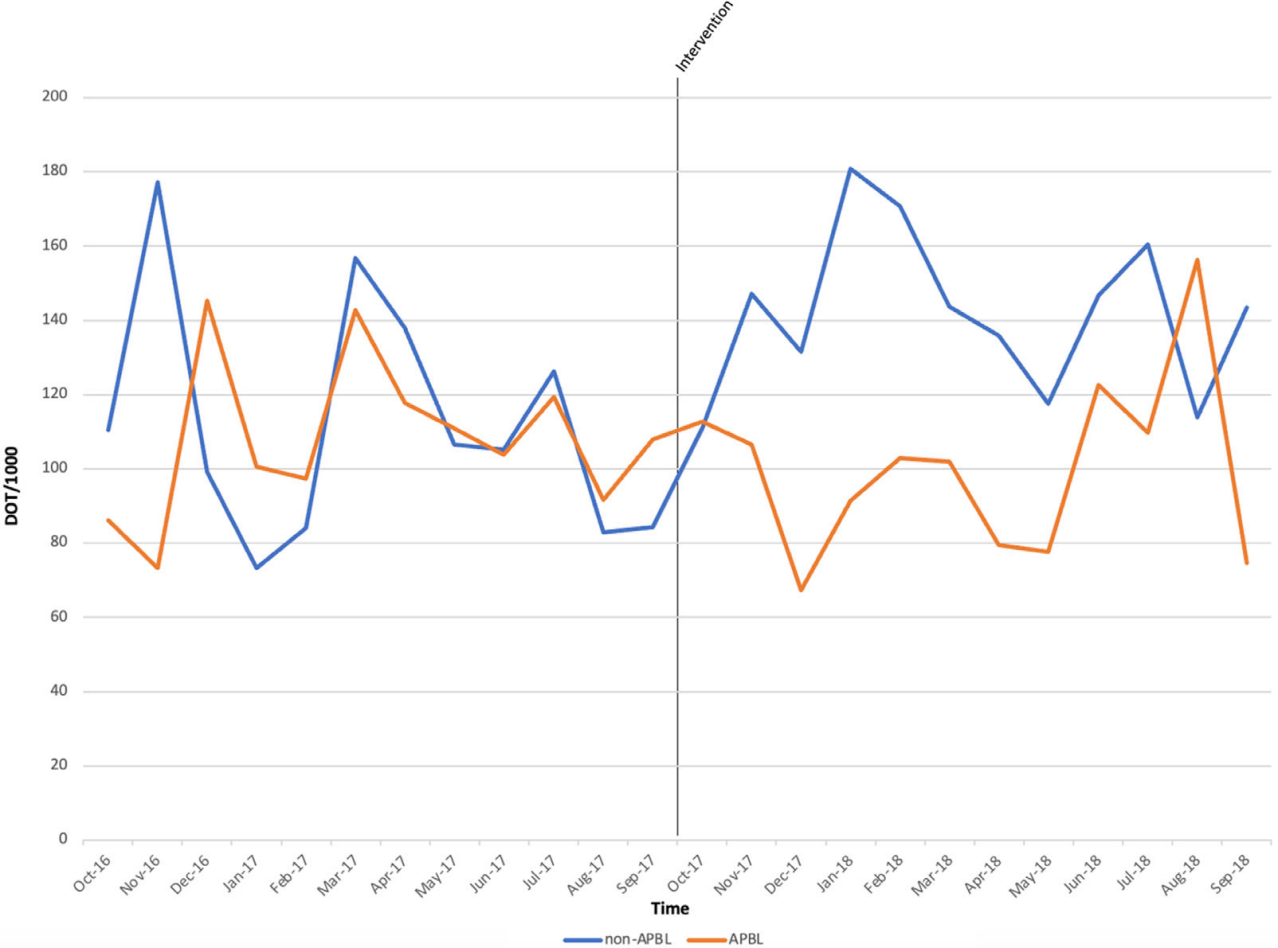
DOT/1000 of APBL and non-APBL in non-ICU settings by month before and our intervention.

A total of 2,562 PAF reviews occurred during the study period. The distribution of syndromes reviewed by PAF were 439 (17%) entries for SSTI, 347 (14%) for UTI, 362 (14%) for cIAI, 338 (13%) for CAP, 136 (5%) for P-AMR, and 931 (37%) for other. By syndrome, APBLs were most used for cIAI (26%), followed by SSTI (25%), P-AMR (15%), CAP (11%), and UTI (8%), while 15% of APBL use did not fit these syndromes. There were 388 stewardship recommendations, of which 239 (62%) were for guideline discordance, 98 (25%) de-escalation, 27 (7%) conversion to PO, 18 (5%) ineffective therapy, and 6 (1%) for end of therapy. Hospitalwide DOT/1000 increased from 355.2 to 376 after 12 months. Days of therapy per 1,000 for APBLs decreased from 52 to 47, and non-APBL use increased from 49 to 57. As a proportion of total beta-lactam use, APBLs decreased from 38% to 32% of total DOT, a 15.79% reduction. In the intensive care unit (ICU), monthly APBL DOT/1000 mean increased from 129 to 206 and non-APBL from 50 to 158 (Figure 1). Mean non-APBL DOT/1000 per month increased in non-intensive care settings (non-ICUs) from 111.98 to 141.85 per month, and APBL had a trend toward reduction from 108 to 100 (Figure 2). The proportion of oral antimicrobials increased from 29% to 35%, a 20.69% increase. Oral AU increased in medical-surgical units from 14% to 30%, a 114.29% increase. Expenditures decreased by 23%, from $172,897 to $132,053, with a cost reduction in APBL of 20% (from $31,674 to $25,389). *C. difficile* infection cases decreased from 24 to 18 cases and rates decreased by 21%, from 3.27 to 2.56 per 10,000 patient days. The antibiogram reported 83 PSAR isolates before our intervention and 108 after. *Pseudomonas aeruginosa* susceptibilities improved for APBLs, with carbapenems improving from 82% (68) to 91% (98), piperacillin–tazobactam from 75% (62) to 89% (96), and cefepime from 82% (68) to 91% (98).

## Discussion

We successfully implemented a syndrome-based stewardship intervention in a safety-net community hospital with limited resources. Our intervention targeted five common infectious syndromes and, though DOT/1000 slightly increased, AU patterns changed by increasing non-APBL use and oral conversion while decreasing APBL use and cost. These changes reflect greater guideline adherence, favoring non-APBL use in most scenarios and APBL use for critical illness or healthcare-associated infections. The increase in AU likely reflects the implementation of the sepsis core measure (SEP-1) during this period and adherence to ASP recommendations for sepsis. Our unit-specific trends highlight optimization to meet critical illness. Antipseudomonal beta-lactam use remained high in the ICU, yet a rise in non-APBLs also occurred. Critical illness often warrants APBLs, but PAF may promote culture-directed de-escalation and syndrome-specific selection even when critically ill. A reduction in APBL use was seen in non-ICU settings, where de-escalation may be more feasible. These trends led to a decrease in antimicrobial expenditures of 23% including a 20% reduction in APBL costs.^8^ Formulary restrictions have successfully reduced APBL use and resistance in ICU settings.^10^ Our syndrome-based intervention had an impact hospitalwide, in accord with other studies in community hospitals.^8^

The decline in CDI and AMR at our facility can have an impact at the patient level and the hospital system. Both can contribute to higher length of stay, costs, and mortality.^1^ *C. difficile* infection can also affect quality scores and value-based purchasing. Thus, the patient safety benefits and hospital savings are important to community hospitals and the populations they serve. Hospitals with <200 beds play a crucial role in healthcare delivery. Safety-net community hospitals serve vulnerable populations and can be financially vulnerable. Our program required no capital expenditures and can be easily replicated in other community hospitals. Our initiative met and exceeded new TJC requirements and led to certification as an ASP Center of Excellence by IDSA.

## Supporting information

Mena Lora et al. supplementary material Mena Lora et al. supplementary material

## Supplementary material

For supplementary material accompanying this paper visit http://doi.org/10.1017/ash.2024.28. click here to view supplementary material
