# Supplementary material for: Impact of a syndrome-based stewardship intervention on antipseudomonal beta-lactam use, antimicrobial resistance, C. difficile rates, and cost in a safety-net community hospital
Source: Antimicrob Steward Healthc Epidemiol. 2024 Mar 13;4(1):e31. doi: 10.1017/ash.2024.28 (PMC10945933; doi:10.1017/ash.2024.28)

**SUPPLEMENTS**

Supplement A. Sample syndrome-based guideline for physicians


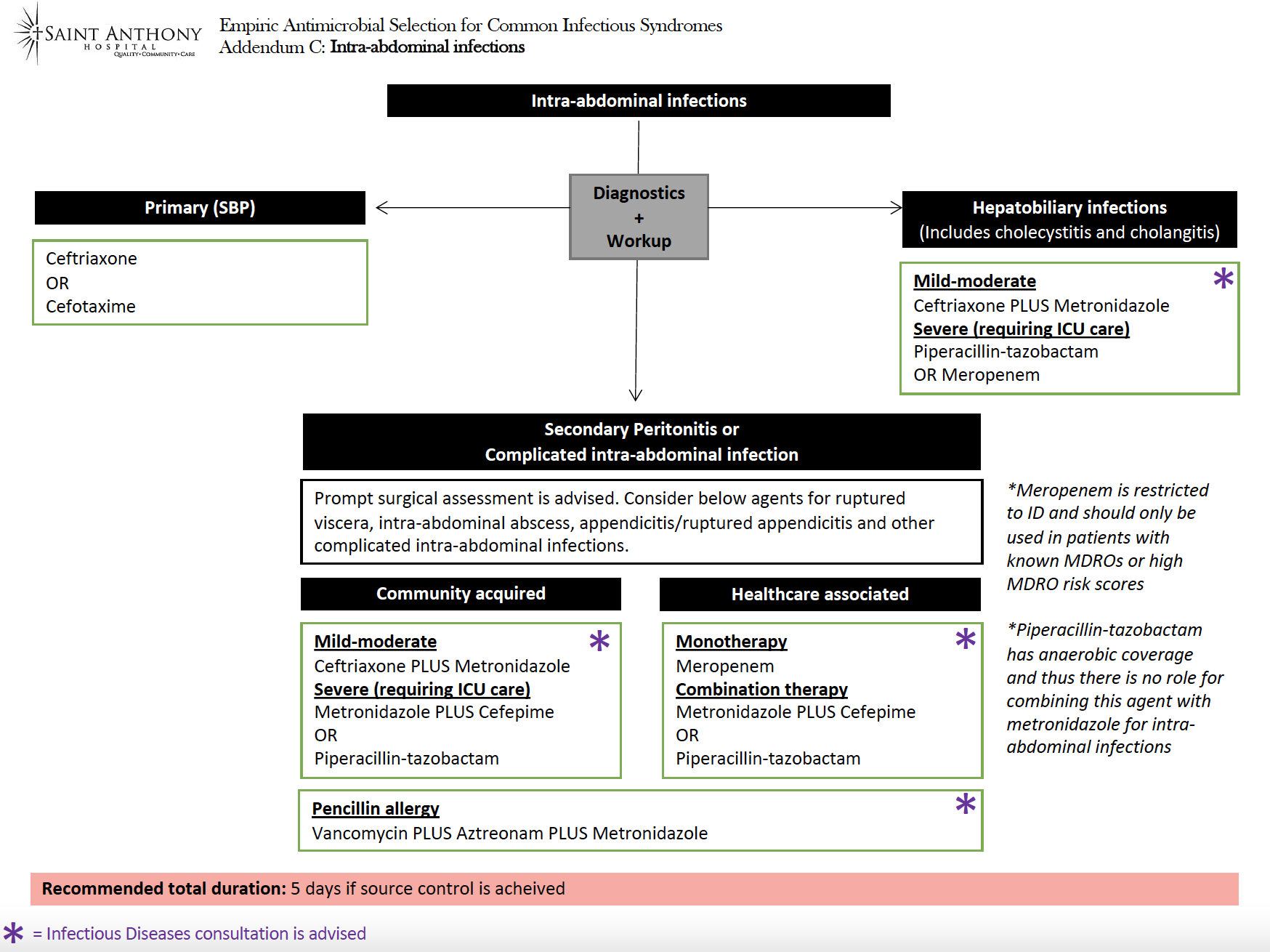


Supplement B. Sample syndrome-based order set for physicians


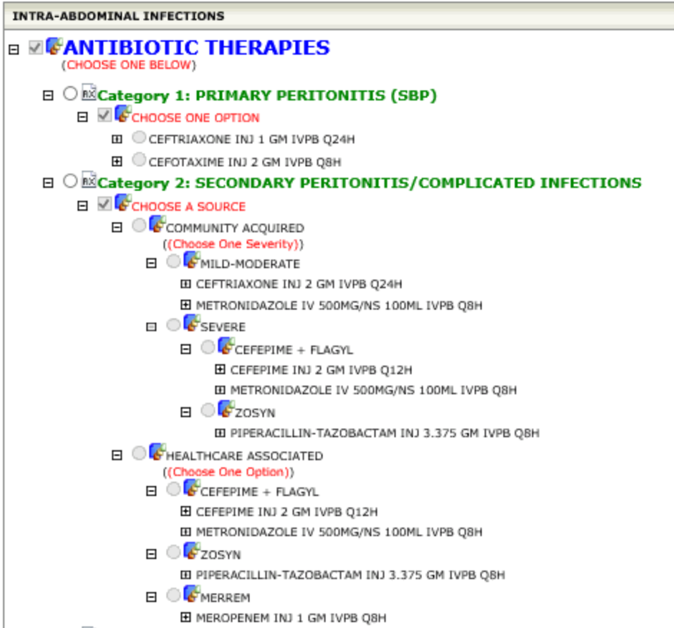

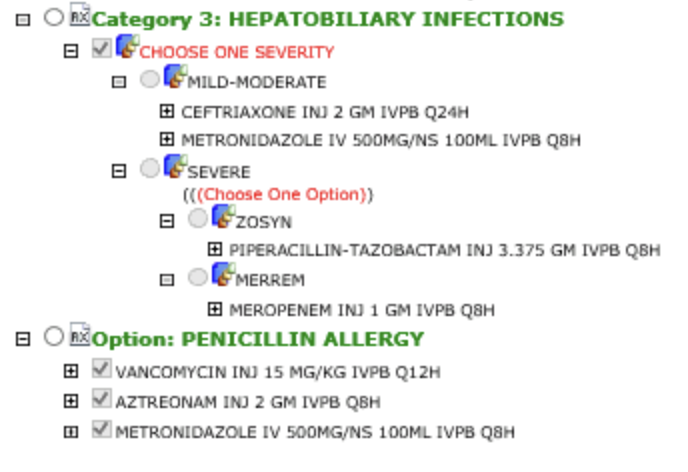


Supplement C. Sample syndrome-based order set for pharmacists


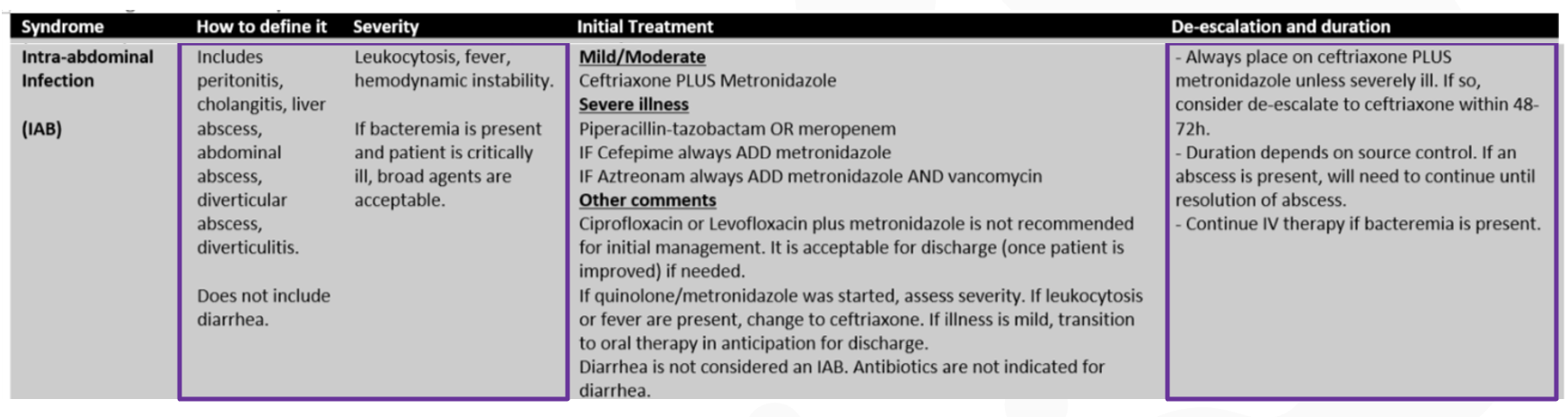

Supplement: Mena Lora et al. supplementary material [file S2732494X24000287sup001.docx]
